# Racial and ethnic disparities in endovascular treatment outcomes in acute ischemic stroke: a systematic review and meta-analysis

**DOI:** 10.1007/s00415-025-13427-z

**Published:** 2025-10-13

**Authors:** Hesham Kelani, Mohamed A. Elzayat, Ahmed Naeem, Hamza Khelifa, Khaled Elbarbary, Daniel Newman, Bara M. Hammadeh, Omar Elsayed Rageh, Amira A. Alghazali, Fatma Mohammed, Mennatullah A. Shehab, Emina Dzafic, Volodymyr Vulkanov, Harneel Saini, David Rosenbaum-Halevi, David P. Lerner, Ernest J. Barthélemy, Fawaz Al-Mufti

**Affiliations:** 1 Department of Neurology, SUNY Downstate Health Sciences University at One Brooklyn Health, Brooklyn, NY USA; 2 Faculty of Medicine, Mansoura University, Mansoura, Egypt; 3 Al-Azhar Faculty of Medicine, Asyut, Egypt; 4 Faculty of Medicine, University of Oran 1, Ahmed Ben Bella, Oran, Algeria; 5 Touro College of Osteopathic Medicine, Harlem, NY USA; 6 Faculty of Medicine, Al-Balqa’ Applied University, Salt, Jordan; 7 Faculty of Medicine, Tanta University, Tanta, Egypt; 8 Faculty of Medicine, Aswan University, Aswan, Egypt; 9 Faculty of Medicine, Cairo University, Cairo, Egypt; 10 Department of Neurology, Rutgers New Jersey School of Medicine, Newark, NJ USA; 11 Department of Neurology, Grand View Hospital, Sellersville, PA USA; 12 Division of Neurosurgery, Department of Surgery, One Brooklyn Health-Brookdale University Hospital, Brooklyn, NY USA; 13 Global Neurosurgery Laboratory, Department of Surgery, SUNY Downstate Health Sciences University, Brooklyn, NY USA; 14 Departments of Neurology, Neurosurgery and Radiology, Westchester Medical Center at New York Medical College, Valhalla, NY USA

**Keywords:** Acute ischemic stroke, Endovascular, Race, Ethnicity, Disparities, White, Black, Hispanic

## Abstract

**Objective:**

This meta-analysis aims to evaluate whether racial and ethnic disparities exist in outcomes following endovascular therapy (EVT) for acute ischemic stroke (AIS).

**Methods:**

A systematic literature search was conducted through June 2024. We used Review Manager to pool data and calculate odds ratios (ORs) for categorical outcomes and mean differences (MDs) for continuous outcomes, all reported with 95% confidence intervals (CIs). Our primary outcomes of interest were functional recovery and mortality 90 days after stroke.

**Results:**

Eleven studies involving 49,040 patients were included. Compared to non-Hispanic patients, Hispanic patients had significantly higher odds of poor functional recovery (mRS 3–6) at 90 days (OR: 1.54; 95% CI 1.20–1.98; P < 0.01), though mortality and sICH rates were similar. When comparing White and non-White patients, White patients had significantly higher 90-day mortality (OR: 1.36; 95% CI 1.15–1.60; P < 0.01), with no significant differences in sICH, recanalization success, or long-term functional recovery.

**Conclusions:**

Disparities in EVT outcomes for AIS appear to be driven more by post-procedural and systemic factors than by differences in the procedure itself. Hispanic patients face worse functional recovery despite similar acute outcomes, suggesting barriers in post-stroke care. Improved access to rehabilitation and culturally tailored support may help close these gaps.

**Supplementary Information:**

The online version contains supplementary material available at 10.1007/s00415-025-13427-z.

## Introduction

Stroke remains the second leading cause of death worldwide and is among the top three causes of death and disability combined [1]. Stroke includes a range of cerebrovascular conditions, but acute ischemic stroke (AIS) is by far the most common, occurring more frequently than hemorrhagic stroke [2]. Endovascular therapy (EVT) has become the gold standard for treating AIS and has played a major role in improving outcomes and reducing long-term disability [3]. However, despite expanded access to EVT and a general decline in stroke-related mortality, racial and ethnic disparities in stroke outcomes continue to persist.

Racial and ethnic disparities in stroke incidence, treatment access, and outcomes have been well-documented in the United States. Black Americans, for example, face a higher stroke incidence rate compared to White Americans and often experience worse outcomes [4–7]. While differences in risk factors like hypertension and diabetes contribute, much of the disparity is driven by social determinants of health, such as limited access to primary and preventive care, less insurance coverage, and delays in recognizing stroke symptoms [8–10]. Hispanic Americans also have higher stroke rates than their White counterparts, with contributing factors that may include language barriers, lower health literacy, and challenges related to immigration status [11]. They also tend to experience longer treatment delays and receive evidence-based treatments like mechanical thrombectomy or tPA less often, particularly in under-resourced communities [12–17].

Although prior research has focused on disparities in stroke incidence and access to acute treatment, few studies have examined whether these disparities affect outcomes after EVT. Given the evolution EVT towards becoming the standard of care for AIS due to large vessel occlusion (LVO), it is important to assess whether all racial and ethnic groups are benefiting equally from its use. The aim of this meta-analysis is to evaluate whether racial and ethnic disparities exist in outcomes following EVT for AIS. Specifically, we compare safety outcomes such as mortality and hemorrhage, and efficacy outcomes like recanalization rates and functional recovery across different racial and ethnic groups. We hope to clarify whether disparities persist even when patients have access to advanced stroke interventions and inform efforts to close equity gaps.

## Methods

### Study protocol

The protocol for this systematic review was registered on PROSPERO (registration number: CRD420251050809). The methodological framework and reporting strategy followed the Preferred Reporting Items for Systematic Reviews and Meta-Analyses (PRISMA) statement guidelines [18, 19].

### Search strategy

For this research, we expanded our search to four major databases: Cochrane Central Register of Clinical Trials (CENTRAL), MEDLINE via PubMed, Scopus, and Web of Science (WOS) to identify studies evaluating racial differences in endovascular treatment outcomes in patients with acute ischemic stroke. The search strategy merged Medical Subject Headings (MeSH) and keywords associated with “acute ischemic stroke”, “endovascular treatment”, and “races and ethnicities”. The searches were carried out in June 2024. The detailed search strategies for each database can be found in the supplementary materials. In addition, further manual searches in reference lists and grey literature were carried out to ensure comprehensive coverage.

### Eligibility criteria

We used the PECOS framework to formulate our research question as follows:

P (Patients): adults aged 18 years or more diagnosed with acute ischemic stroke and treated with endovascular therapy.

E (Exposure): Race (e.g., White, Black) or Ethnicity (e.g., Hispanic or Latino).

C (comparator): Different race or ethnicity. The study should include at least two racial or ethnic groups to be included in this analysis.

O (Outcomes): safety outcomes, including 90-day mortality, symptomatic intracranial hemorrhage (sICH), and any intracranial hemorrhage and efficacy outcomes, including successful recanalization rate (defined as Thrombolysis in Cerebral Infarction (TICI) score ≥ 2b), Excellent functional outcomes (defined as mRS 0–1) at 90 days, and poor functional outcomes (defined as mRS 3–6).

Study design: retrospective or prospective cohort studies, and case–control studies.

Studies with no racial or ethnic comparisons, case reports, narrative reviews, or of inadequate methodological quality were excluded. In addition, non-English-language studies were excluded.

### Study selection

Records retrieved from the database search were first added to EndNote to remove duplicates, then uploaded to Rayyan software to start the title and abstract screening. Two authors independently assessed the titles and abstracts for eligibility. Potentially eligible articles identified by title and abstract screening were further evaluated using their full text by two authors. Disagreements were resolved by consensus or, if necessary, consultation with the first author.

### Data extraction

A standardized data extraction sheet was developed using Google Sheets, incorporating key study characteristics, interventions, and outcomes. Two independent authors reviewed the sheet before pilot testing. Then, three randomly selected studies were used to assess the clarity and consistency, and feedback was incorporated to refine the final version. Data extraction was conducted independently by two authors. Disagreement was resolved by consensus or, if necessary, consultation with the first author.

### Risk of bias assessment

Two independent authors used the Risk Of Bias In Non-randomized Studies—of Exposures (ROBINS-E) to assess the risk of bias in seven domains: confounding, selection of participants, classification of exposures, deviations from intended exposures, missing data, measurement of outcomes, and selection of reported results [20]. Similar to other steps, disagreements were resolved by consensus or, if necessary, consultation with the first author. Results of the risk of bias assessment were presented using traffic light plots and weighted bar plots generated by the Robvis tool [21].

### Data synthesis and analysis

All extracted data were pooled and analyzed using Review Manager software (RevMan 5.4). Given the limited availability of sufficiently granular data for each racial and ethnic subgroup, race was dichotomized as White versus non-White, and ethnicity as Hispanic versus non-Hispanic. Dichotomous variables were pooled to estimate the odds ratio (OR), while continuous variables were pooled to calculate the mean difference (MD). The results were reported with a 95% confidence interval (CI). Modified Rankin scale scores were categorized as: Excellent (0–1), good (0–2) and poor (3–6) functional recovery as reported by previous studies. As good and poor are complementary, we only calculated odds ratios for excellent and poor functional recovery. The I^2^ statistic was employed to assess statistical heterogeneity. A fixed-effects model was used for studies with low statistical heterogeneity, while a random-effects model was applied for those with high heterogeneity (I^2^ > 40%). All results were assumed to be statistically significant at the p < 0.05 level.

## Results

### Selection of included studies

**Fig. 1:**
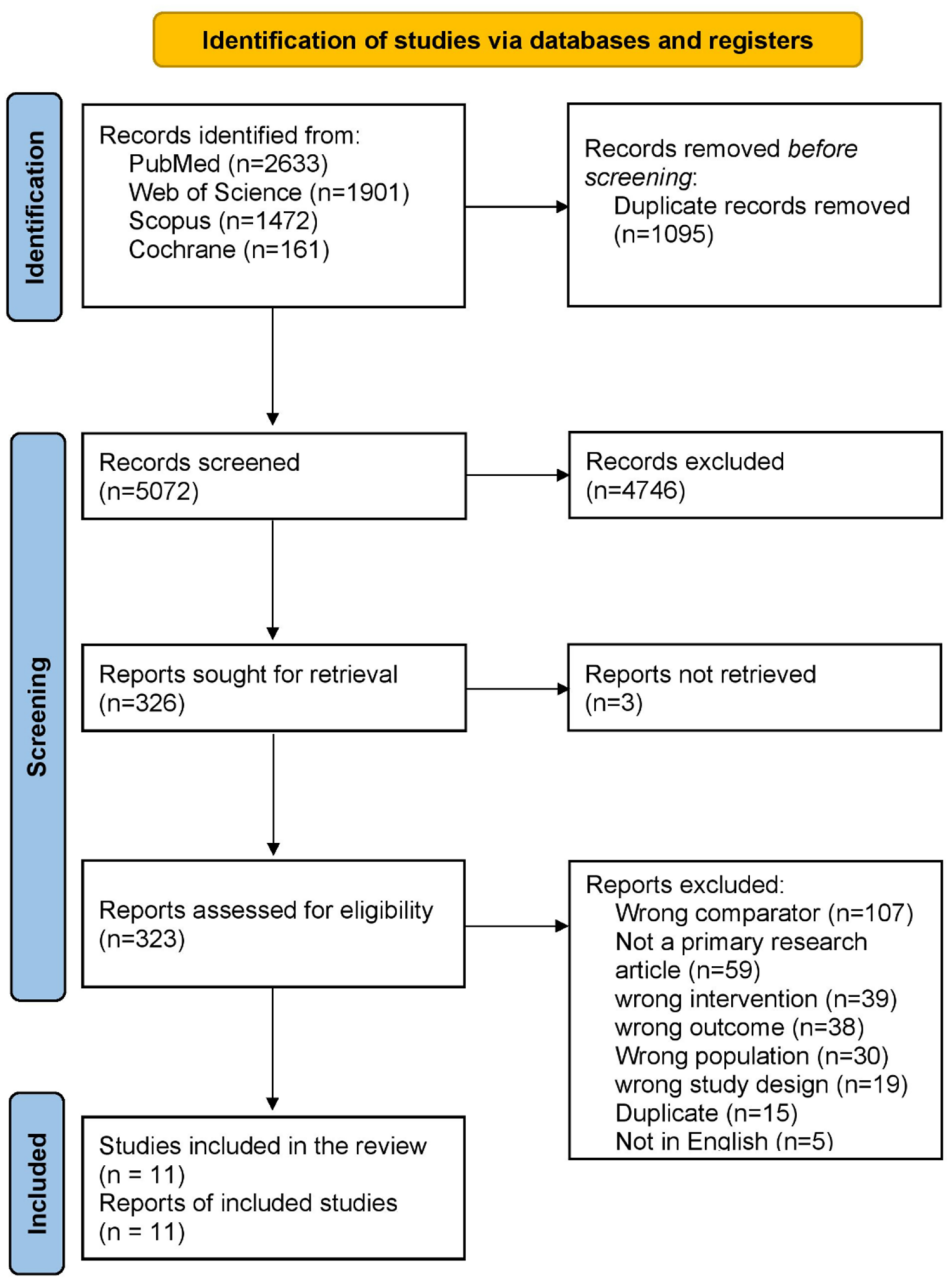
PRISMA flowchart

Using our search strategy, we initially identified 6167 publications. Duplicate records were detected and removed, leaving 5072 publications for screening. These articles then underwent title and abstract screening, followed by full-text screening. Finally, we detected 11 eligible studies that were included in this systematic review [22–32] (Fig. 1).

### Study characteristics

Eleven studies, encompassing a total of 49,040 participants, with sample sizes ranging from 157 to 42,422 patients, were included in the systematic review. All studies were retrospective cohort studies, and most of them were conducted in the USA. A detailed summary of the included studies is presented in Table 1.

**Table 1:** Summary of included studies

Study ID	Study design	country	Data source	Total number of participants	Distribution of races/ethnicities (percent)	EVT techniques	Primary outcome(s)	sICH definition
Chiang et al., 2019	Retrospective cohort	USA	UC San Diego Stroke Center’s Institutional Review Board-approved prospective stroke registry	157	White: 127 (80.89) Black: 13 (8.28) Asian: 13 (8.28) Other: 4 (2.55)	NA	Rates of symptomatic intracerebral hemorrhage (sICH) and major systemic hemorrhage (MSH)	Defined based on NINDS criteria: Any worsening in neurological status with hemorrhage confirmed on CT scan
Sheriff et al., 2022 [23]	Retrospective cohort	USA	Get With the Guidelines-Stroke (GWTG-Stroke) database	42,422	NHW 29 429 (69.4) NHB 6214 (14.6) Hispanic 2887 (6.8) Asian 1342 (3.2) Other 2550 (6.01)	NA	Temporal Trend (pre- and post-2015) in EVT utilization and outcomes according to race/ethnicity	–
Bouslama et al., 2018 [30]	Retrospective cohort	USA	Grady Endovascular Stroke Outcomes Registry	616	White: 308 (50%) Black: 308 (50%)	stent retrievers White 67.2%, Black 59.7%	90-day modified Rankin Scale (mRS)	–
Catapona et al., 2021 [29]	Retrospective cohort	USA	institutional dataset from St. Joseph’s Hospital and Medical Center (Barrow Neurological Institute, Phoenix, AZ)	401	Black 28 (7.0%) White 373 (93.0%)	NA	Hospital length of stay, mortality, NIHSS score, and the mRS score at the last follow-up	NA
Fuentes et al., 2023	Retrospective cohort	USA	NeuroVascular Quality Initiative-Quality Outcomes Database (NVQI-QOD) registry, a multicenter database involving 28 U.S. centers across 17 states	1522	NHW: 761 NHB: 761^a^	NA	Post procedure TICI score, post procedure length of stay, in-hospital mortality, discharge to home, discharge NIHSS score, discharge mRS score, 90-day mRS score, and 90-day mortality	–
Jones et al., [27]	Retrospective cohort	USA	stroke program registry of the authors’ institute	666	NHW 300 (45) NHB 197 (29) Hispanic 123 (19)^b^	NA	90-day modified Rankin Scale (mRS)	Defined based on SITS-MOST criteria
Mohammaden et al., 2022 [26]	Retrospective cohort	USA	prospectively maintained database of patients with acute ischemic stroke treated with mechanical thrombectomy from October 2010 through June 2020 to identify all consecutive patients with age ≥ 80 years and anterior circulation large vessel occlusion strokes	344	White 251(73) Black 93 (27)	NA	90-day modified Rankin Scale (mRS)	parenchymal hematoma leading to neurological deterioration, as reflected by NIHSS score worsening of ≥ 4 points
Salhader et al., 2021 [25]	Retrospective cohort	NA	a comprehensive stroke center dataset that was prospectively collected between 2012 and 2020	215	Hispanic 139(64.7) non-Hispanic 76 (35.3)	NA	90-day modified Rankin Scale (mRS)	NA
Samuels et al., 2020 [24]	Retrospective cohort	Northern New Zealand	Northern Region component of the New Zealand Stroke Registry	256	Māori 46 (17.97) Pacific 32 (12.50%) Asian 27 (10.55%) NZ European/other 151 (58.98%)	NA	90-day mRS	–
Burks et al., 2021 [22]	Retrospective cohort	North America and Europe	the STAR (Stroke Thrombectomy and Aneurysm Registry) database	2115	NHW 1535 (72.58) NHB 295 (13.95) Hispanic 285 (13.48)	Aspiration first 22.6%, 21%, 19.6% Stent–retreiver first 41%, 46.1, 48.1% Solumbra first 22.5%, 8.1%, 8.1% Other 13.8%, 24.7%, 24.2%^c^	90-day modified Rankin Scale (mRS)	defined using ECAS III (European Cooperative Acute Stroke Study) definition (worsening of ≥ 4 points in National Institutes of Health Stroke Scale attributed to hemorrhagic transformation)
Srinivas et al., 2023	Retrospective cohort	USA	Two tertiary care facilities between 2019 and 2022	326	NHB 137 (42) NHW 173 (53) non-Hispanic Other 16 (4.9)	Stent retriever: NHB 39.4%, NHW 40.5%, Other 6 37.5%	Modified Rankin Scale (mRS) score, mortality, and discharge disposition at 3, 6, and 12 months following thrombectomy	–

a: We included the sub-sample from propensity score matching to control for confounding variables

b: (7%) Patients with no race/ethnicity data or listed as other racial groups were not included in our meta-analysis

c: The percentages are for NHW, NHB, and Hispanic, respectively

Of the 49,040 participants, 33,257 were White, while 8046, 3477 were Black and Hispanic, respectively. Regarding the White group, 16,397 (49.49%) patients were female. The mean age ranged from 63.0 ± 16.6 years to 85 ± 4.47 years. Furthermore, 13,110 (39.42%) received IVT together with MT. On the other hand, 4032 (50.15%) patients were female in the Black group. The mean age ranged from 61.1 ± 15.4 years to 84.67 ± 4.52 years. Three thousand five hundred seventy-four (44.43%) received IVT as a part of their care (Table 2).

**Table 2:** Baseline characteristics of the included studies

Study ID		N (%)	Age, years, (mean ± SD)	Sex (Female) N (%)	Baseline NIHSS, (mean ± SD)	ASPECTS median (IQR)	occlusion site					
							ICA	ACA	MCA	PCA	Vertebra-basilar	Tandem occlusion
Chiang et al., 2020 [31]	All sample	157 (100)	NA	NA	NA	NA	NA	NA	NA	NA	NA	NA
	White	127 (80.89)	NA	NA	NA	NA	NA	NA	NA	NA	NA	NA
	Black	13 (8.28)	NA	NA	NA	NA	NA	NA	NA	NA	NA	NA
	Asian	13 (8.28)	NA	NA	NA	NA	NA	NA	NA	NA	NA	NA
	others	4 (2.55)	NA	NA	NA	NA	NA	NA	NA	NA	NA	NA
	Hispanic	43(27.39)	NA	NA	NA	NA	NA	NA	NA	NA	NA	NA
	Non-Hispanic	114 (72.61)	NA	NA	NA	NA	NA	NA	NA	NA	NA	NA
Sheriff et al., 2022 [23]	All sample	42,422 (100)	71.5 ± 2.4	21,634 (51.0)	17.7 ± 1.07	NA	NA	NA	NA	NA	NA	NA
	Hispanic	2887 (6.8)	69.5 ± 3.12	1,411 (48.9)	18 ± 1.42	NA	NA	NA	NA	NA	NA	NA
	White	29,429 (69.4)	73.5 ± 2.4	15,081 (51.2)	17.2 ± 1.09	NA	NA	NA	NA	NA	NA	NA
	Black	6214 (14.6)	64 ± 2.9	3,148 (50.7)	17.75 ± 1.2	NA	NA	NA	NA	NA	NA	NA
	Asian	1342 (3.2)	71.7 ± 2.8	707 (52.7)	18.5 ± 1.5	NA	NA	NA	NA	NA	NA	NA
Bouslama et al., 2018 [30]	All sample	616 (100)	63.1 ± 13.46	280 (45.5)	18.32 ± 5.9	7.5 [6.5 – 8.5]	109 (17.7)	5 (0.8)	366 (59.4)	NA	59 (9.6)	77 (12.5)
	Caucasians	308 (50)	64.68 ± 12.75	135 (43.8)	18.04 ± 5.42	7 [6–8]	48 (15.6)	1 (0.3)	173 (56.2)	NA	32 (10.4)	54 (17.5)
	African Americans	308 (50)	61.51 ± 13.98	145 (47.1)	18.59 ± 6.34	8 [7–9]	61 (19.8)	4 (1.3)	193 (62.7)	NA	27 (8.8)	23 (7.5)
Catapono et al., 2021 [29]	All sample	401 (100)	68.3 ± 13.5	168 (41.9)	15.2 ± 7.5	NA	161 (40.1)^a^	5 (1.2)^b^	205 (51.1)	NA	27 (6.7)	18 (4.5)
	White	373 (93.02)	68.9 ± 13.2	161 (43.2)	15.1 ± 7.5	NA	148 (39.7)^a^	4 (1.1)^b^	191 (51)	NA	27 (7.3)	18 (4.9)
	Black	28 (7)	61.1 ± 15.4	7 (25)	15.2 ± 8.2	NA	13 (46.4)^a^	1 (3.6)^b^	14 (50)	NA	0 (0)	Zero
Fuentes et al., 2023	All sample	1522 (100)	62.6 ± 16.4	729 (47.9)	16.5 ± 7.4	NA	334 (21.9)	10 (0.7)	1036 (68.1)	6 (0.4)	22 (1.4)	NA
	NHW	761 (81)	63.0 ± 16.6	358 (47.0)	16.7 ± 7.4	NA	195 (25.6)	6 (0.8)	485 (63.7)	3 (0.4)	11 (1.4)	NA
	NHB	761 (19)	62.1 ± 16.2	371 (48.8)	16.3 ± 7.4	NA	139 (18.3)	4 (0.5)	551 (72.4)	3 (0.4)	11 (1.4)	NA
Jones et al., 2021 [27]	All sample	666 (100)	66.3 ± 9	292 (44)	17 ± 7	8.0 (7,10)	102 (15.3)	NA	462 (69.4)	NA	69 (10)	24 (4)
	NHW	300 (45)	69.8 ± 7	138 (46)	17 ± 7	8.0 (7,10)	40 (13)	NA	223 (74)	NA	24 (8)	9 (3)
	NHB	197 (29)	62.3 ± 10	90 (46)	17 ± 7.0	9 (7,10)	37 (19)	NA	131 (67)	NA	21 (11)	6 (3)
	Hispanic	123 (19)	63.5 ± 10	55 (41)	17 ± 7	8 (7,10)	13 (11)	NA	83 (68)	NA	16 (13)	8 (7)
Mohammaden et al., 2022 [26]	All sample	344 (100)	85 ± 4.4	239 (69.5)	9 ± 5.9	9 (7–9)^e^	73 (21.2)	1 (0.3)	270 (78.4)	NA	NA	19 (5.5)
	White	251 (73)	85 ± 4.47	173 (68.9)	19 ± 5.96	9 (7, 9)^f^	58 (23.1)	1 (0.4)	192 (76.5)	NA	NA	15 (6)
	African American	93 (27)	84.67 ± 4.52	66 (71)	18.67 ± 6.78	9 (8–10)^g^	15 (16.1)	1 (0.4)	78 (38.9)	NA	NA	4 (4.3)
Salhader et al., 2021	All sample	215 (100)	NA	132 (61.4)	18.12 ± 8.3	NA	41 (19.1)	6 (2.8)	161 (74.9)	NA	14 (6.5)	NA
	Hispanic	139 (64.6)	NA	86 (61.9)	19 ± 8.89	NA	21 (15.1)	4 (2.9)	107 (77)	NA	8 (5.8)	NA
	Non-Hispanic	76 (35.3)	NA	46 (60.5)	16.5 ± 6.67	NA	20 (26.3)	2 (2.6)	54 (71.1)	NA	6 (7.9)	NA
Samuels et al., 2020 [24]	All sample	256 (100)	67.9 ± 14.9	97 (38)	15.33 ± 7.46	NA	NA	NA	NA	NA	NA	NA
	Māori	46 (17.9)	59 ± 14	22 (48)	14.50 ± 7.6	NA	NA	NA	NA	NA	NA	NA
	Pacific	32 (12.5)	57.1 ± 15.6	9 (28)	16.33 ± 7.76	NA	NA	NA	NA	NA	NA	NA
	Asian	27 (10.5)	68 ± 12.8	12 (44)	18.00 ± 3.91	NA	NA	NA	NA	NA	NA	NA
	NZ European/other	151 (58.9)	73 ± 12.8	54 (36)	14.67 ± 6	NA	NA	NA	NA	NA	NA	NA
Burks et al., 2021 [22]	All sample	2115 (100)	69.83 ± 14.8	1033 (48.8)	15.91 ± 7.4	NA	444 (21)	10 (0.5)	1437 (67.9)	31 (1.5)	193 (9.1)	Zero
	NHW	1535 (73)	70.67 ± 15.58	751 (48.9)	16 ± 7.42	9 (8–10)	330 (21.5)	9 (0.6)	1027 (66.9)	20 (1.3)	149 (9.7)	Zero
	NHB	295 (14)	63.33 ± 14.15	136 (46.1)	15.67 ± 8.19	9 (8–10)	68 (23.1)	Zero	192 (65)	7 92.4)	28 (9.5)	Zero
	Hispanic	285 (13)	72 ± 8.84	146 (51.2)	15.67 ± 6.71	10 (8–10)	46 (16.1)	1 (0.4)	218 (76.5)	4 (1.4)	16 (5.7)	Zero
Srinivas et al., 2023	All sample	326 (100)	66.61 ± 15.8	177 (54.2)	14.96 ± 7.45	NA	17 (5.2)	6 (1.8)	252 (77.3)	36 (11.04)		NA
	NHW	173 (53.1)	70.3 ± 15.3	96 (55.5)	15.0 ± 6.7	NA	12 (6.2)	2 (1)	137 (70.2)	13 (6.7)^h^		NA
	NHB	137 (42)	62.3 ± 14.7	69 (50.4)	15.0 ± 8.2	NA	5 (3.2)	4 (2.5)	104 (66.2)	20 (12.7)^h^		NA
	Other	16 (4.9)	63.6 ± 21.9	12 (75)	14.2 ± 8.8	NA	Zero	Zero	11 (68.8)	3 (18.8)^h^		NA

Study ID		Preexisting medical conditions									Time interval mean (SD)		Length of hospital stay in days (mean ± SD)	IV Thrombolysis N (%)
		Diabetes	HTN	smoking	HF	AF	CAD/MI	Dyslipidemia	previous TIA	previous ischemic stroke	Onset/presentation to groin	Groin to recanalization		
Chiang et al., 2020 [31]	All sample	NA	NA	NA	NA	NA	NA	NA	NA	NA	NA	NA	NA	72 (45.9)
	White	NA	NA	NA	NA	NA	NA	NA	NA	NA	NA	NA	NA	61 (48)
	Black	NA	NA	NA	NA	NA	NA	NA	NA	NA	NA	NA	NA	6 (46.2)
	Asian	NA	NA	NA	NA	NA	NA	NA	NA	NA	NA	NA	NA	5 (38.5)
	others	NA	NA	NA	NA	NA	NA	NA	NA	NA	NA	NA	NA	0 (0)
	Hispanic	NA	NA	NA	NA	NA	NA	NA	NA	NA	NA	NA	NA	24 (55.8)
	Non-Hispanic	NA	NA	NA	NA	NA	NA	NA	NA	NA	NA	NA	NA	48 (42.1)
Sheriff et al., 2022 [23]	All sample	10,556 (24.9)	30,110 (71.0)	6,983 (16.5)	5,664 (13.4)	15,066 (35.5)	10,134 (23.9)	17,879 (42.1)	9,078 (21.4)		NA	NA	NA	16,969 (40.0)
	Hispanic	1,043 (36.1)	2,034 (70.5)	291 (10.1)	303 (10.5)	889 (30.8)	620 (21.5)	1,101 (38.1)	628 (21.8)		NA	NA	NA	1,329 (46.1)
	White	6,566 (22.3)	20,654 (70.2)	4,856 (16.5)	3,729 (12.7)	11,268 (38.3)	7,428 (25.2)	12,998 (44.2)	6,136 (20.9)		NA	NA	NA	11,330 (38.5)
	Black	1,953 (31.4)	4,739 (76.3)	1,366 (22.0)	1,236 (19.9)	1,571 (25.3)	1,334 (21.5)	2,237 (36.0)	1,542 (24.8)		NA	NA	NA	2,817 (45.3)
	Asian	358 (26.7)	957 (71.3)	94 (7.0)	111 (8.3)	490 (36.5)	235 (17.5)	566 (42.2)	290 (21.6)		NA	NA	NA	618 (46.1)
Bouslama et al., 2018 [30]	All sample	156 (25.3)	461 (74.8)	129 (20.9)	NA	200 (32.5)	NA	236 (38.3)	NA	NA	361.00 ± 191.09	76.59 ± 44.1	NA	274 (44.5)
	Caucasians	67 (21.8)	211 (68.5)	69 (22.4)	NA	100 (29.9)	NA	111 (36)	NA	NA	366.67 ± 158.65	74.50 ± 42.8	NA	137 (44.8)
	African Americans	89 (28.9)	250 (81.2)	60 (19.5)	NA	100 (29.9)	NA	125 (40.6)	NA	NA	355.33 ± 218.9	78.67 ± 45.4	NA	137 (44.8)
Catapono et al., 2021 [29]	All sample	117 (29.2)	293 (73.1)	102 (25.4)	NA	NA	168 (41.9)	162 (40.4)^c^	NA	72 (18)	50.5 ± 49.6^d^	42.0 ± 29.3	8.5 (8.2)	176 (43.9)
	White	109 (29.2)	275 (73.7)	90 (24.1)	NA	NA	157 (42.1)	146 (39.1)^c^	NA	68 (18.2)	51.3 ± 50.1	43.0 ± 29.2	8.6 ± 8.4	167 (44.8)
	Black	8 (28.6)	18 (64.3)	12 (42.9)	NA	NA	11 (39.3)	16 (57.1)^c^	NA	4 (14.3)	38.6 ± 40.5	27.3 ± 27.0	7.2 ± 4.9	9 (32.1)
Fuentes et al., 2023	All sample	516 (33.9)	1237 (81.3)	460 (30.2)	256 (16.8)	364 (23.9)	254 (16.7)	564 (37.1)	NA	347 (22.8)	NA	NA	10.1 ± 14.1	594/1518 (39.1)
	NHW	268 (35.2)	627 (82.4)	231 (30.4)	132 (17.3)	183 (24.0)	130 (17.1)	284 (37.3)	NA	171 (22.5)	NA	NA	9.1 ± 10.0	300/758 (39.6)
	NHB	248 (32.6)	610 (80.2)	229 (30.1)	124 (16.3)	181 (23.8)	124 (16.3)	280 (36.8)	NA	176 (23.1)	NA	NA	11.2 ± 17.2	294/760 (38.7)
Jones et al., 2021 [27]	All sample	167 (32)	407 (76)	74 (15)	NA	151 (28)	NA	251 (47)^c^	NA	75 (14)	103 ± 56	NA	6.33 ± 5.2	360 (54)
	NHW	53 (22)	187 (78)	40 (18)	NA	87 (36)	NA	104 (44)^c^	NA	31 (13)	95 ± 46	NA	6 ± 4.47	162 (54)
	NHB	54 (33)	127 (77)	23 (15)	NA	40 (24)	NA	78 (47)^c^	NA	29 (18)	110 ± 59	NA	7.67 ± 5.97	108 (55)
	Hispanic	49 (46)	75 (71)	8 (8)	NA	19 (18)	NA	53 (50)^c^	NA	13 (12)	111 ± 60	NA	6.67 ± 6	71 (58)
Mohammaden et al., 2022 [26]	All sample	83 (24.1)	282 (82)	20 (5.8)	NA	203 (59)	NA	130 (37.8)	NA	NA	377.00 ± 254.6	59.67 ± 45.4	NA	124 (36)
	White	54 (21.5)	198 (78.9)	17 (6.8)	NA	162 (64.5)	NA	93 (37.1)	NA	NA	322 ± 248.15	58.67 ± 43.99	NA	93 (37.1)
	African American	29 (31.2)	84 (90.3)	3 (3.2)	NA	41(44.1)	NA	37 (39.8)	NA	NA	357 ± 272.59	65.42 ± 47.63	NA	31 (33.3)
Salhader et al., 2021	All sample	80 (37.2)	193 (89.8)	7 (3.3)	28 (13.0)	106 (49.3)	59 (27.4)	113 (52.6)^c^	41 (19.1)	NA	98.21 ± 63.6	36.06 ± 24.2	6.00 ± 4.9	73 (34.0)
	Hispanic	59 (42.4)	131 (94.2)	5 (3.6)	15 (10.8)	62 (44.6)	37 (26.6)	72 (51.8)^c^	31 (22.3)	NA	94.5 ± 65.5^d^	35 ± 22.22	6 ± 5.19	45 (32.4)
	Non-Hispanic	21 (27.6)	62 (81.6)	2 (2.6)	13 (17.1)	44 (57.9)	22 (28.9)	41 (53.9)^c^	10 (13.2)	NA	105 ± 60^d^	38 ± 27.41	6 ± 4.44	28 (36.8)
Samuels et al., 2020 [24]	All sample	NA	NA	NA	NA	NA	NA	NA	NA	NA	234.67 ± 118.54	NA	NA	NA
	Māori	NA	NA	NA	NA	NA	NA	NA	NA	NA	213.67 ± 49.74	NA	NA	NA
	Pacific	NA	NA	NA	NA	NA	NA	NA	NA	NA	220.33 ± 86.92	NA	NA	NA
	Asian	NA	NA	NA	NA	NA	NA	NA	NA	NA	209.33 ± 83.75	NA	NA	NA
	NZ European/other	NA	NA	NA	NA	NA	NA	NA	NA	NA	240.00 ± 125	NA	NA	NA
Burks et al., 2021 [22]	All sample	602 (28.5)	1582 (74.8)	NA	NA	750 (35.5)	NA	894 (42.3)^c^	NA	NA	283.67 ± 210.5	45.66 ± 31.8	NA	1052 (49.7)
	NHW	385 (25.1)	1114 (72.6)	NA	NA	600 (39.1)	NA	685 (44.6)^c^	NA	NA	279.33 ± 184.76	47.67 ± 33.39	NA	799 (52.1)
	NHB	100 (33.9)	235 (79.7)	NA	NA	63 (21.4)	NA	100 (33.9)^c^	NA	NA	314 ± 294.27	41.00 ± 25.33	NA	122 (41.4)
	Hispanic	117 (41.1)	233 (81.8	NA	NA	87 (30.59)	NA	109 (38.2)^c^	NA	NA	275.67 ± 233.96	39.67 ± 27.57	NA	131 (46)
Srinivas et al., 2023	All sample	110 (33.7)	281 (86.2)	118 (36.2)	NA	137 (42)	139 (42.6)	NA	NA	37 (11.3)	162.35 ± 184.9	48.58 ± 53.37	12.35 ± 13.8	117 (35.9)
	NHW	49 (28.3)	148 (85.5)	60 (34.7)	NA	82 (47.4)	81 (46.8)^i^	NA	NA	20 (11.6)	156.8 ± 189.6	49.7 ± 64.9	10.2 ± 8.6	61 (35.5)
	NHB	56 (40.9)	122 (89.1)	56 (40.9)	NA	49 (35.8)	53 (38.7)^i^	NA	NA	17 (12.4)	175.9 ± 186.3	46.2 ± 34.8	15.1 ± 18	50 (36.5)
	Other	5 (7)	11 (68.8)	2 (12.5)	NA	6 (37.5)	5 (31.3)^i^	NA	NA	0	106.4 ± 95.5	56.8 ± 47.9	12.1 ± 15.7	6 (37.5)

a: MCA/ICA, Cervical ICA, Intracranial ICA, Tandem ICA, ACA/ICA

b: ACA/ICA

c: hyperlipidemia

d: The time interval here is admission to puncture

e: n = 318

f: n = 232

g: n = 86

h: Posterior circulation

i: this includes patients with coronary artery disease, prior myocardial infarction, and congestive heart failure

### Risk of bias assessment

**Fig. 2:**
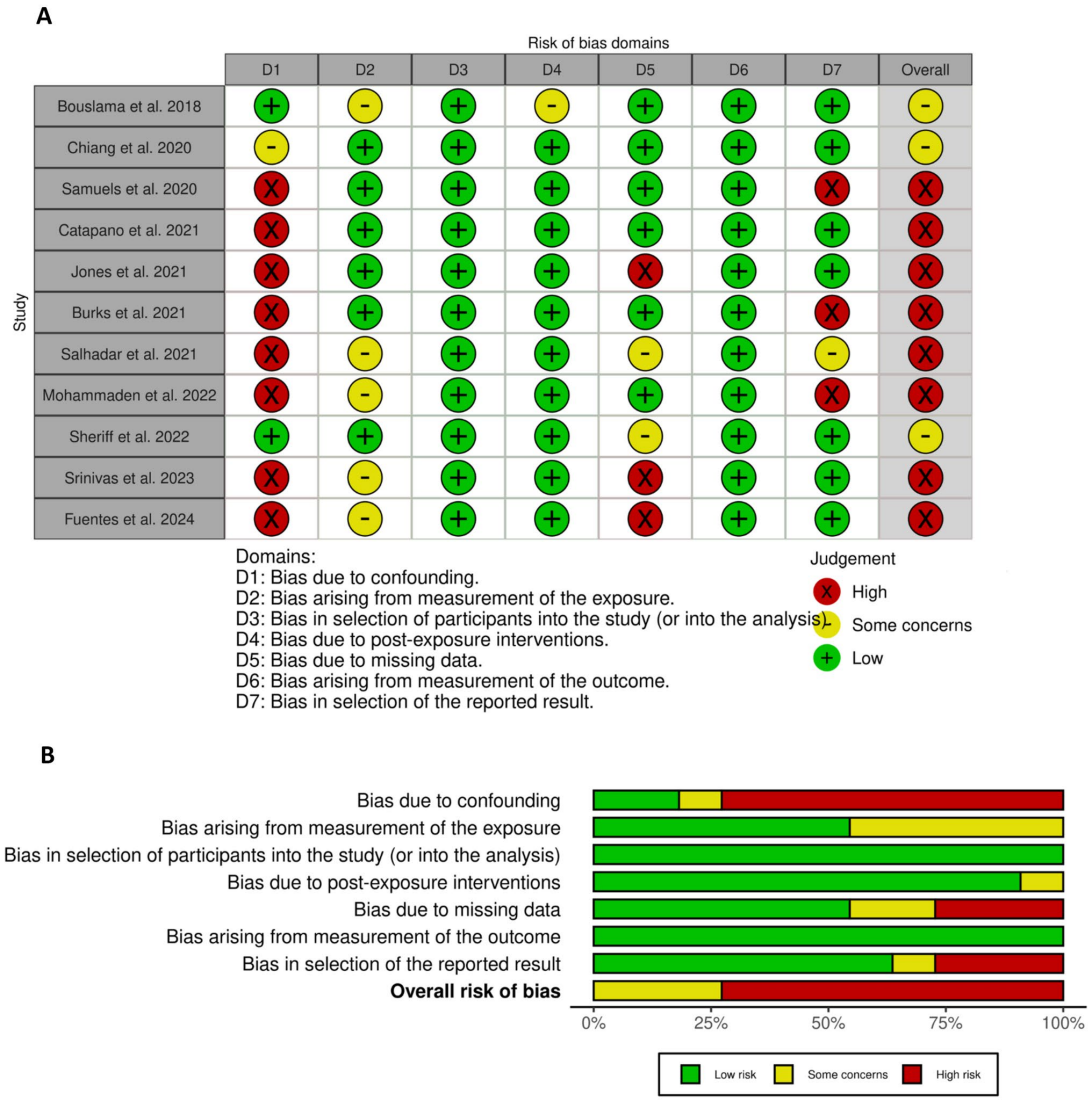
Risk of bias assessment using ROBINS-E. **A** Traffic Light Plot of bias assessment. **B** summary plot of risk of bias assessment. Green–Low risk, yellow–some concerns, red- high risk

Most of the included studies had an overall high risk of bias. Only three studies had some concerns [23, 30, 31]. Furthermore, all studies showed a low risk of bias arising from outcome measurement, as well as bias in the selection of participants. Only one study was found to raise some concerns regarding bias due to post-exposure intervention [30], whereas the remaining studies demonstrated a low risk of bias (Fig. 2).

### Data analysis

#### Hispanic vs non-hispanic

##### Safety outcomes

**Fig. 3:**
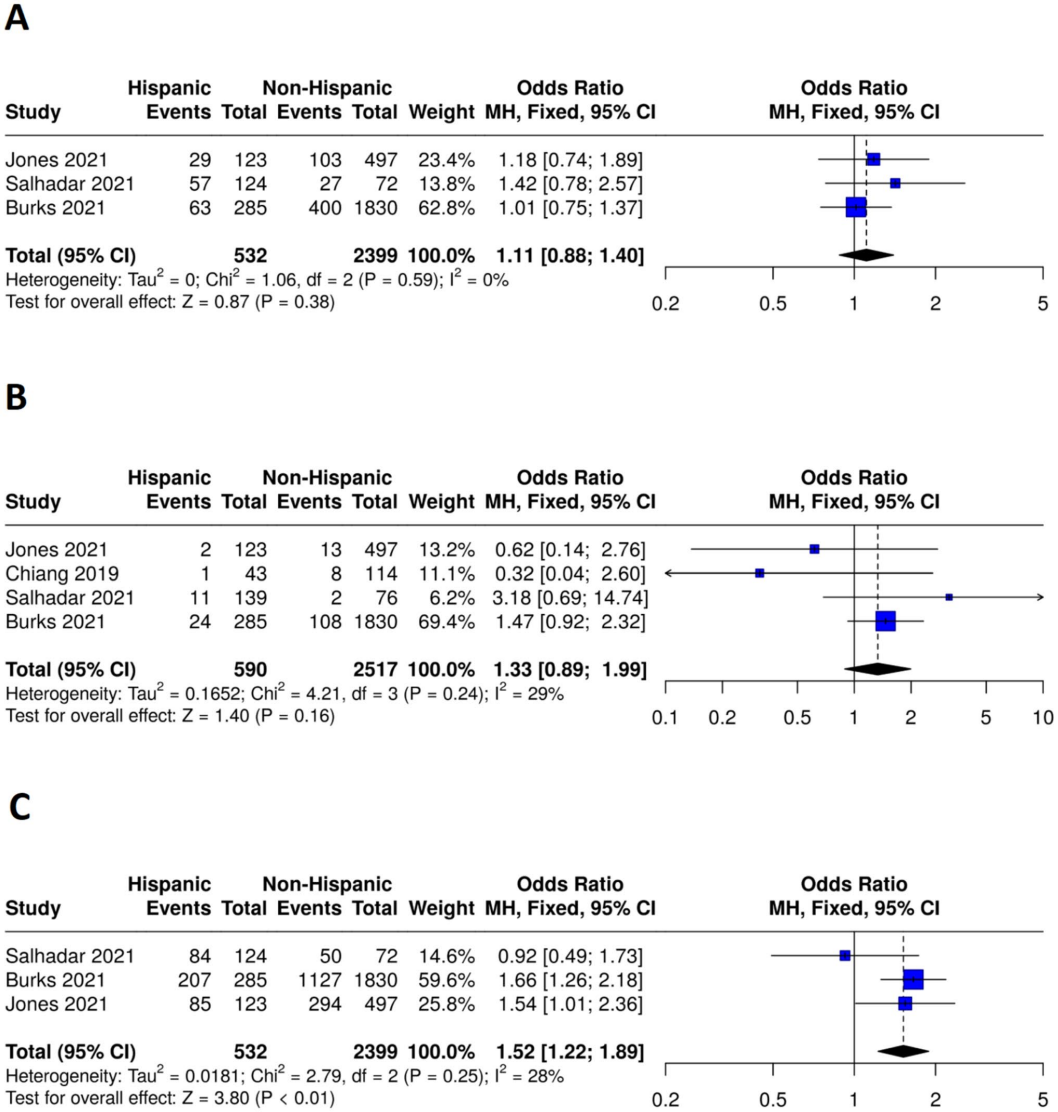
Forest plots comparing safety and efficacy outcomes between Hispanic and non-Hispanic patients receiving endovascular therapy (EVT) for acute ischemic stroke. **A** Mortality, **B** Symptomatic intracranial hemorrhage, **C** poor functional recovery at 90 days(mRS 3–6)

*Mortality:* The pooled analysis for mortality showed no statistically significant difference between the Hispanic (events = 149, total = 532) and non-Hispanic (events = 530, total = 2399) groups, with an overall odds ratio (OR) of 1.09 (95% CI: 0.89–1.34, P = 0.38). The analysis revealed no heterogeneity among the studies (I^2^ = 0%, P = 0.59), indicating consistent results across the included studies as presented in Fig. 3A.

*Symptomatic intracranial hemorrhage (ICH)*: The meta-analysis for symptomatic intracranial hemorrhage (ICH) demonstrated no statistically significant difference between the Hispanic (events = 38, total = 590) and non-Hispanic (events = 131, total = 2517), with an overall odds ratio (OR) of 1.33 (95% CI: 0.89–1.99, P = 0.16). The analysis revealed low heterogeneity among the studies (I^2^ = 29%, P = 0.24), as presented in Fig. 3B.

##### Efficacy outcomes

*Poor functional recovery*: The Modified Rankin Scale (mRS) three to six at 90 days showed a statistically significant difference between the Hispanic (events = 376, total = 532) and non-Hispanic (events = 1471, total = 2459) groups, with an odds ratio (OR) of 1.54 (95% CI: 1.20–1.98, P < 0.01). The analysis revealed low heterogeneity among the studies (I^2^ = 28%, P = 0.25), as presented in Fig. 3C.

#### White vs non-white

##### Safety outcomes

**Fig. 4:**
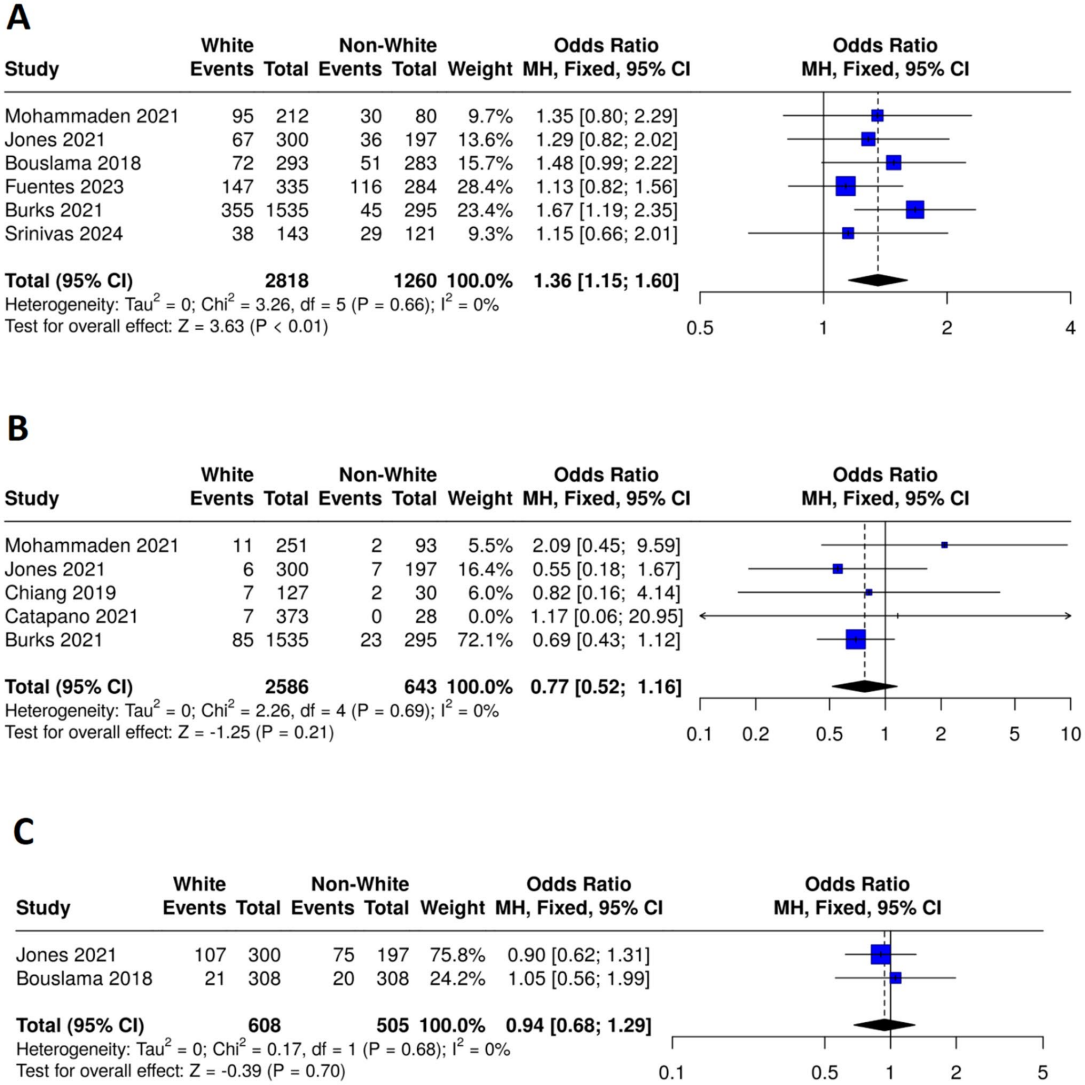
Forest plots comparing safety outcomes between white and non-white patients. **A** Mortality, **B** Symptomatic intracranial hemorrhage, **C** Any intracranial hemorrhage

*Mortality*: Mortality analysis demonstrated a statistically significant difference between the White (events = 774, total = 2818) and non-White (events = 307, total = 1260) groups, with an odds ratio (OR) of 1.36 (95% CI: 1.15–1.60, P < 0.01). The analysis revealed no heterogeneity among the studies (I^2^ = 0%, P = 0.66), as presented in Fig. 4A.

*Intracranial hemorrhage (ICH)*: The meta-analysis for symptomatic intracranial hemorrhage (ICH) showed no statistically significant difference between the White (events = 116, total = 2586) and non-White (events = 34, total = 643) groups, with an odds ratio (OR) of 0.77 (95% CI: 0.52–1.16, P = 0.21) with no heterogeneity (I^2^ = 0%, P = 0.69), as presented in Fig. 4B. Similarly, the meta-analysis for any intracranial hemorrhage (ICH) showed no statistically significant difference between the White (events = 128, total = 608) and non-White (events = 95, total = 505) groups, with an odds ratio (OR) of 0.94 (95% CI: 0.68–1.29, P = 0.70). No heterogeneity was detected among the studies (I^2^ = 0%, P = 0.68), as presented in Fig. 4C.

##### Efficacy outcomes

**Fig. 5:**
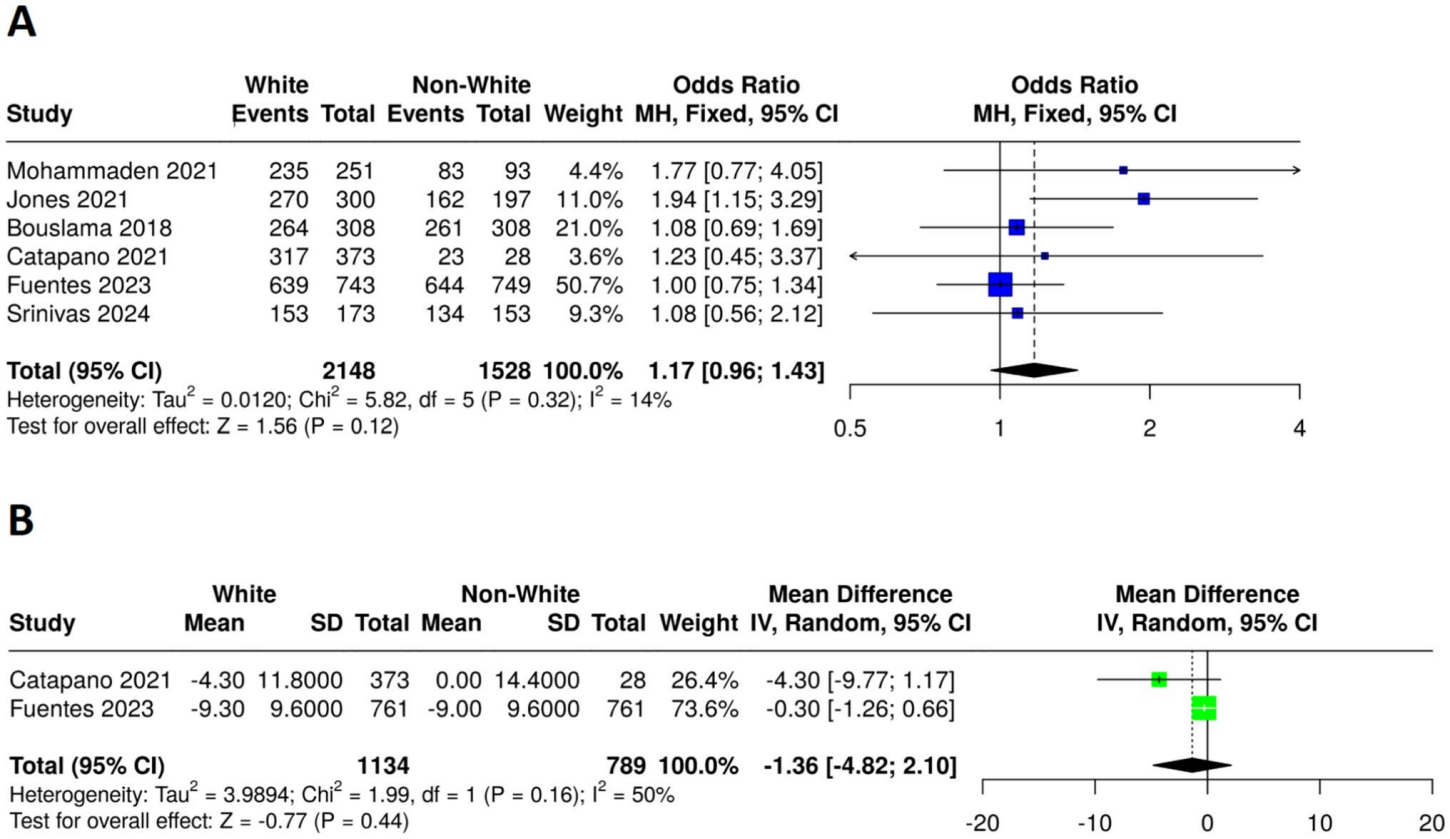
Forest plots comparing efficacy outcomes between white and non-white patients **A** Successful recanalization based on the TICI (2B-3) scale, **B** Mean NIHSS change from admission to discharge

*Successful recanalization*: The pooled analysis for Thrombolysis in Cerebral Infarction (TICI) scale (2b-3) demonstrated no statistically significant difference between the White (events = 1878, total = 2148) and non-White (events = 1307, total = 1528) groups, with an odds ratio (OR) of 1.17 (95% CI: 0.96–1.43, P = 0.12) with low heterogeneity among the studies (I^2^ = 14%, P = 0.32), as presented in Fig. 5A.

*The NIH Stroke Score (NIHSS)*: The NIH Stroke Score (NIHSS) mean change from admission to discharge showed no statistically significant difference between the White and non-White groups, with a mean difference of -0.30 (95% CI: -1.26 to 0.66, P = 0.44). Moderate heterogeneity was observed among the studies (I^2^ = 50%, P = 0.16) as presented in Fig. 5B.

##### The modified rankin scale (mRS)

The Modified Rankin Scale (mRS) results were analyzed at discharge and 90 days after.

**Fig. 6:**
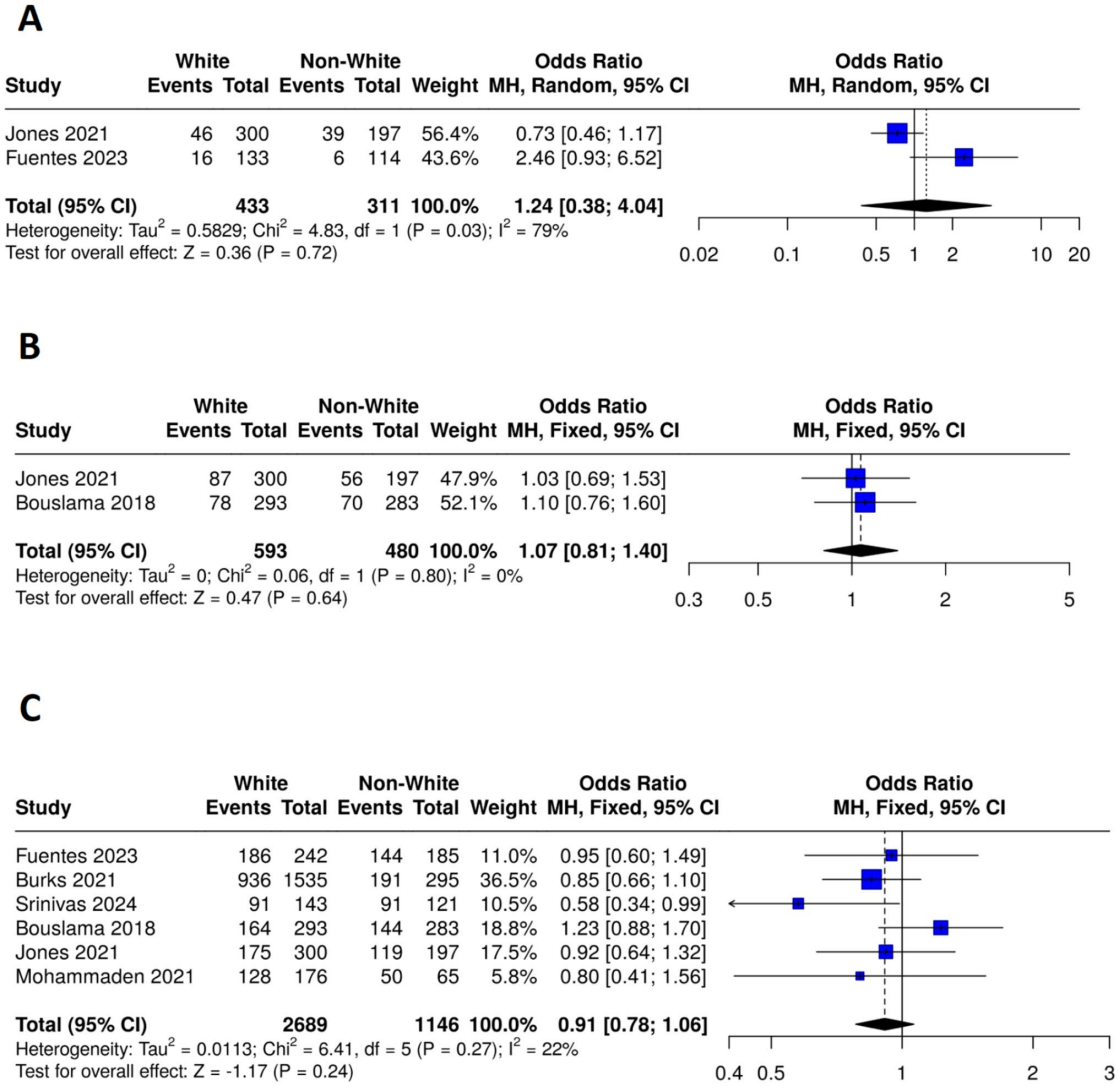
Forest plots comparing functional recovery between white and non-white patients at discharge and 90 days. **A** Excellent recovery at discharge (mRS 0–2), **B** Excellent recovery at 90 days (mRS 0–1), **C** Poor recovery at 90 days (mRS 3–6)

*At discharge*: The mRS (0–2) at discharge analysis demonstrated no statistically significant difference between the White (events = 62, total = 433) and non-White (events = 45, total = 311) groups, with an odds ratio (OR) of 1.24 (95% CI: 0.38–4.04, P = 0.72) with high heterogeneity (I^2^ = 79%, P = 0.03) as presented in Fig. 6A.

*Ninety days after stroke*: The excellent functional recovery (mRS (0–1)) at 90 days of follow-up pooled analysis revealed no statistically significant difference between the White (events = 165, total = 593) and non-White (events = 126, total = 480) groups, with an odds ratio (OR) of 1.07 (95% CI: 0.81–1.40, P = 0.64). The analysis showed no heterogeneity among the studies (I^2^ = 0%, P = 0.80) as presented in Fig. 6B. Similarly, no statistically significant difference was observed in poor functional recovery (mRS (3–6)) between the White (events = 1680, total = 2689) and non-White (events = 739, total = 1146) groups, with an odds ratio (OR) of 0.91 (95% CI: 0.78–1.06, P = 0.24) with low heterogeneity among the studies (I^2^ = 22%, P = 0.27) as presented in Fig. 6C.

## Discussion

Our meta-analysis evaluated racial and ethnic disparities in outcomes following endovascular treatment (EVT) for acute ischemic stroke (AIS). We identified significant differences in functional recovery between Hispanic and non-Hispanic patients and mortality between White and non-White patients.

Our analysis found no significant difference in mortality or symptomatic intracranial hemorrhage between Hispanic and non-Hispanic patients. These findings are consistent with prior literature suggesting that in-hospital stroke care quality tends to be equitable when standardized protocols are followed [33]. Still, even though these results weren’t statistically significant, selection bias might have played a role. The Hispanic patients who did receive EVT may have already overcome significant barriers, so there could still be a gap in care. However, it is encouraging that once access to care is reached, the treatment itself seems consistent across different ethnic groups. On the other hand, Hispanic patients had significantly higher odds of poor functional recovery at 90 days. In comparison to our previously mentioned findings, this potentially indicates a difference in the post-procedure quality of care. This highlights a gap not in the delivery of EVT itself, but with what happens afterwards in recovery. Functional recovery is heavily influenced by several factors after the procedure. First, gaps in insurance coverage can limit access to rehabilitation services and essential medications to promote recovery or prevent progression of other comorbidities. Second, living far from a comprehensive stroke center or rehabilitation facility can make it harder for patients to receive consistent follow-up care. Third, language barriers as well as limited health literacy may interfere with understanding discharge instructions, further increasing the difficulty in adhering to therapy. Taken individually or in combination, these factors may help explain why Hispanic patients face worse functional recovery despite receiving comparable care [34].

Other variables, such as stroke subtype, severity, and location, also influence recovery and may differ across ethnic groups [35]. Our findings underscore the need not only to expand access to EVT but also to make sure that the care Hispanic patients get afterwards meets their needs. If we don’t address these post-procedure issues, then equal access to procedures alone won’t be enough to close the gap in outcomes.

We also found that White patients had significantly higher post-procedural stroke mortality than non-White patients. This trend is similar to most recent studies and demonstrates a shift, as older studies found higher mortality in non-White patients [36, 37]. One explanation is the finding of a previous study that found that White patients are more likely to have cardioembolic strokes, which are associated with more severe neurological deficits. This would increase short-term mortality. Additionally, non-White patients experience strokes at a younger age, which may lead to greater recovery and improved early survival [38]. This finding suggests that race-based differences in stroke outcomes may be more strongly influenced by underlying comorbidities than by disparities in access to care. Furthermore, case selection differences (more aggressive use of EVT in white patients, including in technically challenging or less promising situations) may also contribute to this finding.

There were no significant differences between White and non-White groups in sICH or any ICH. Likewise, there were no significant differences between White and non-White patients in successful recanalization, NIHSS scores mean change from admission to discharge, or the mRS (0–2) at discharge. These results indicate that EVT is performed safely and just as effectively for patients of all racial backgrounds. This points to the underlying cause of racial outcome differences in patients with AIS being treated with EVT being disparities in the social determinants of health influencing access to EVT, and post-treatment care and recovery pathways. Interestingly, and different from the comparison between Hispanic and non-Hispanic patients, excellent functional outcomes and poor functional recovery 90 days after stroke showed no statistically significant differences. This finding may suggest progress toward reducing race-based disparities in recovery from AIS.

This meta-analysis has limitations. First, races and ethnicities were self-defined and didn’t have a standardized definition. Furthermore, we dichotomized races and ethnicities due to insufficient data on each race and ethnicity. This could mask differences between subgroups and constrain the generalizability of our results. In addition, risk of bias was high in many of included studies with controlling for confounders being the domain with highest risk. Subgrouping and sensitivity analyses based on bias level were avoided as it would fragment the already small pool of comparable studies. Confounding variables were the major concern in most studies and unfortunately most studies didn’t report enough data about them and if they tried to control for confounders, so we couldn’t adjust effect estimates based on confounders or conduct a meta-regression. The same issue was observed with treatment modality. Not all participants received the same treatment, with some being treated with EVT while others were treated with IV tPA plus EVT. No study reported separate outcome data based on the treatment modality, so we couldn’t assess each modality separately. Furthermore, most studies have similar percentage of who received IV tPA which was around 40%, so we couldn’t perform subgroup analyses based on this percentage. Due to small number of included studies, we couldn’t assess the publication bias. To consider publication bias, ten or more studies should contribute to an outcome which is not the case in this study. Finally, while heterogeneity was generally low, a few outcomes had moderate-to-high heterogeneity, suggesting variations across the included studies.

Future research should focus on collecting patient-level data that also includes data on social determinants of health such as income, education, and location. Also, prospective studies could explore whether targeted interventions, like improved discharge planning, better access to rehabilitation services, or culturally tailored patient education can reduce the disparities. Overall, our findings suggest that disparities in EVT outcomes are not due to differences in the procedure but are due to disparities in social determinants of health influencing access to the procedure and care pathways after the procedure. The worse functional recovery among Hispanic patients reinforces the need for reforms in both acute stroke interventions and post-care protocols. To start closing these gaps, stroke care system priorities include facilitating access of underserved populations to rehabilitation services, expanding insurance coverage, mitigating language and cultural barriers, and increasing awareness of structural and social determinants of health, stroke care and outcomes in communities that are often underserved.

## Conclusion

Our findings demonstrate that while EVT is performed with similar success and safety across racial and ethnic groups, disparities in post-procedural outcomes persist, particularly for Hispanic patients, who face worse functional recovery despite comparable acute care. These results suggest that the disparities in outcomes are due to systemic barriers in follow-up care and rehabilitation. Moreover, the higher mortality among White patients may reflect differences in stroke subtypes and comorbidities rather than inequities in care access. Focusing on post-stroke care, especially in underserved communities, is essential if for closing these gaps and achieving more equitable stroke outcomes.

## Supplementary Information

Below is the link to the electronic supplementary material.

Supplementary file1 (PDF 201 KB)

## Data Availability

All data generated or analyzed during this work are included in this article and its supplementary materials.
