# Supplementary material for: Racial and ethnic disparities in endovascular treatment outcomes in acute ischemic stroke: a systematic review and meta-analysis
Source: J Neurol. 2025 Oct 13;272(10):691. doi: 10.1007/s00415-025-13427-z (PMC12518384; doi:10.1007/s00415-025-13427-z)
Supplement: Supplementary file 1 — Supplementary file1 (PDF 201 KB) [file 415_2025_13427_MOESM1_ESM.pdf]

Racial And Ethnic Disparities In Endovascular Treatment Outcomes In Acute Ischemic Stroke: A Systematic Review And Meta-Analysis

Hesham Kelani[1⁋], Mohamed A. Elzayat[2⁋*], Ahmed Naeem[3], Hamza Khelifa[4], Khaled Elbarbary[2], Daniel Newman[5], Bara M. Hammadeh[6], Omar Elsayed Rageh[7], Amira A. Alghazali[2], Fatma Mohammed[8], Mennatullah A. Shehab[9], Emina Dzafic[5], Volodymyr Vulkanov[10*], Harneel Saini[11], David Rosenbaum-Halevi[1], David P Lerner[1], Ernest J. Barthélemy[1,12,13], Fawaz Al-Mufti[14].

1. Department of Neurology, SUNY Downstate Health Sciences University at One Brooklyn Health, Brooklyn, NY.
2. Faculty of Medicine, Mansoura University, Mansoura, Egypt.
3. Al-Azhar Faculty of Medicine, Asyut, Egypt.
4. Faculty of Medicine, University of Oran 1, Ahmed Ben Bella, Oran, Algeria.
5. Touro College of Osteopathic Medicine, Harlem, NY.
6. Faculty of Medicine, Al-Balqa' Applied University, Salt, Jordan.
7. Faculty of Medicine, Tanta University, Tanta, Egypt.
8. Faculty of Medicine, Aswan University, Aswan, Egypt.
9. Faculty of Medicine, Cairo University, Cairo, Egypt.
10. Department of Neurology, Rutgers New Jersey School of Medicine, Newark, NJ.
11. Department of Neurology, Grand View Hospital, Sellersville, PA.
12. Division of Neurosurgery, Department of Surgery, One Brooklyn Health-Brookdale University Hospital, Brooklyn, NY.
13. Global Neurosurgery Laboratory, Department of Surgery, SUNY Downstate Health Sciences University, Brooklyn, NY.
14. Departments of Neurology, Neurosurgery and Radiology, Westchester Medical Center at New York Medical College, Valhalla, NY

⁋ Hesham Kelani and Mohamed A. Elzayat contributed equally to this work.

*Correspondence to (Volodymyr Vulkanov; vv263@njms.rutgers.edu and Mohamed A. Elzayat; m.elzayat08@gmail.com)

Cochrane:

#1 "cerebrovascular disorders" OR "basal ganglia cerebrovascular disease" OR "brain ischemia" OR "carotid artery diseases" OR "carotid artery thrombosis" OR "carotid artery, internal, dissection" OR "intracranial arterial diseases" OR "cerebral arterial diseases"

#2 "anterior cerebral artery infarction" OR "middle cerebral artery infarction" OR "posterior cerebral artery infarction" OR "infarction, anterior cerebral artery" OR "infarction, middle cerebral artery" OR "infarction, posterior cerebral artery"

#3 "Lacunar Infarct" OR "Lacunar Infarction" OR "intracranial embolism" OR "intracranial thrombosis" OR "brain infarction" OR "vertebral artery dissection"

#4 ((brain OR cerebr* OR cerebell* OR vertebrobasil* OR hemispher* OR intracran* OR intracerebral OR infratentorial OR supratentorial OR middle cerebr* OR anterior circulation) near/5 (Ischemi* OR Ischaemi* OR infarct* OR thrombo* OR emboli* OR occlus* OR hypoxi*))

#5 ((Ischemi* OR Ischaemi*) near/6 (stroke* OR apoplex* OR cerebral vasc* OR cerebrovasc* OR cva OR attack*))

#6 "cerebral sinus thrombosis" OR "cerebral venous sinus thrombosis" OR "CVST" OR "CVT"

#7 #1 OR #2 OR #3 OR #4 OR #5 OR #6

#8 "interventional radiology" OR catheterization OR "balloon catheterization" OR angioplasty OR "laser-assisted balloon angioplasty" OR "laser angioplasty" OR atherectomy OR "catheter ablation" OR embolectomy OR "aspiration embolectomy" OR "balloon embolectomy"

#9 thrombectomy OR "mechanical thrombectomy" OR neurothrombectom* OR "thrombus aspiration" OR "blood vessel prosthesis" OR "blood vessel transplantation" OR "cerebral revascularization" OR reperfusion OR dilatation

#10 angioplast* OR stent* OR atherect* OR thromboaspiration OR endoluminal repair* OR endovascular snare* OR neuronet OR microsnare OR angiojet OR "penumbra system" OR "solitaire" OR "trevo"

#11 (interventional NEAR/3 (radiolog* OR radiograph* OR neuroradiolog*))

#12 ((mechanical OR radiolog* OR pharmacomechanical OR laser OR endovascular OR neurovascular) NEAR/5 (thrombolys* OR reperfusion OR fragmentation OR aspiration OR recanalisation OR recanalization OR clot lysis OR clot lyses))

#13 ((clot OR thrombus OR thrombi OR embol*) NEAR/5 (aspirat* OR remov* OR retriev* OR fragmentation OR retract* OR extract* OR obliterat* OR dispers*))

#14 ((retrieval OR extraction) NEAR/5 device*)

#15 (("blood vessel" OR artery) NEAR/5 (prosthesis OR implantat*))

#16 ((merci OR concentric) NEAR/5 retriever)

#17 ((Endovascular OR intravascular) NEXT (Procedure* OR Technique* OR treatment*))

#18 #8 OR #9 OR #10 OR #11 OR #12 OR #13 OR #14 OR #15 OR #16 OR #17

#19 ethnic* OR race OR races OR racial OR Nationalit* OR foreign* OR "Minority groups" OR "Ethnic groups" OR Multilingualism OR "Population groups" OR "Continental population groups"

#20 "Hispanic Americans" OR "African Continental Ancestry Group" OR "American native continental ancestry group" OR "Asian Continental ancestry group" OR "European Continental Ancestry Group" OR "Oceanic ancestry group" OR "African Americans" OR Arabs

#21 "Asian Americans" OR "Mexican Americans" OR Inuits OR Jews OR "Indians, South American" OR "Indians, North American" OR "South American Indians" OR "North American Indians" OR "Cultural Characteristics"

#22 migrant* OR immigrant* OR asylum OR indig* OR aborig* OR native* OR inuit* OR eskimo* OR kalaallit* OR amerind* OR romany OR romanies OR gypsies OR gipsies OR black OR blacks OR asian OR latin* OR Indian)

#23 #19 OR #20 OR #21 OR #22

#24 #7 AND #18 AND #23

PubMed:

#1 "Ischemic Stroke"[Mesh] OR stroke OR "acute stroke*" OR "ischemic stroke" OR "acute ischemic stroke" OR " Embolic Stroke" OR " Thrombotic Stroke" OR "cerebrovascular Accident*" OR "cerebral vascular accident*" OR "Brain Vascular Accident*" OR "cerebrovascular disorders" OR "basal ganglia cerebrovascular disease" OR "thrombotic events" OR "thromboses"

#2 "acute cerebral ischemia" OR "cerebral ischemia" OR "brain ischemia" OR "cerebral infarction" OR "brain infarction" OR "Lacunar Infarct" OR "Lacunar Infarction" OR "intracranial embolism" OR "intracranial thrombosis" OR "vertebral artery dissection"

#3 "carotid artery diseases" OR "carotid artery thrombosis" OR "carotid artery, internal, dissection" OR "intracranial arterial diseases" OR "cerebral arterial diseases" OR "cerebrovascular apoplex*"

#4 "cerebral arterial occlusion" OR "cerebral artery occlusion" OR "anterior cerebral artery infarction" OR "middle cerebral artery infarction" OR "posterior cerebral artery infarction" OR "infarction, anterior cerebral artery" OR "infarction, middle cerebral artery" OR "infarction, posterior cerebral artery" OR "cerebral sinus thrombosis" OR "cerebral venous sinus thrombosis" OR "CVST" OR "CVT"

#5 #1 OR #2 OR #3 OR #4

#6 "Endovascular Procedures"[Mesh] OR "endovascular Procedure*" OR "endovascular Technique*" OR "endovascular treatment*" OR "endovascular therap*"

#7 angioplasty OR "laser-assisted balloon angioplasty" OR "laser angioplasty" OR atherectomy OR "catheter ablation" OR embolectomy OR "aspiration embolectomy" OR "balloon embolectomy" OR "interventional radiology" OR catheterization OR "balloon catheterization"

#8 thrombectomy OR "mechanical thrombectomy" OR "mechanical thrombolysis" OR "neurothrombectom*" OR "thrombus aspiration" OR "blood vessel prosthesis" OR "blood vessel transplantation" OR "cerebral revascularization" OR reperfusion OR dilatation OR angioplast* OR stent* OR atherect* OR thromboaspiration OR endoluminal repair* OR endovascular snare* OR neuronet OR microsnare OR angiojet OR "penumbra system" OR "solitaire" OR "trevo"

#9 "Retrieval device"[Title/Abstract:~5] OR "Retrieval devices"[Title/Abstract:~5] OR "extraction device"[Title/Abstract:~5] OR "extraction devices"[Title/Abstract:~5] OR "blood vessel prosthesis"[Title/Abstract:~5] OR "blood vessel implantation"[Title/Abstract:~5] OR "artery prosthesis"[Title/Abstract:~5] OR "artery implantation"[Title/Abstract:~5]OR "merci retriever"[Title/Abstract:~5] OR "concentric retriever"[Title/Abstract:~5]

#10 #6 OR #7 OR ##8 OR #9

#11 ethnic* OR race OR races OR racial OR Nationalit* OR foreign* OR "Minority groups" OR "Ethnic groups" OR Multilingualism OR Refugees OR "Population groups" OR "Continental population groups"
#12 "Hispanic Americans" OR "African Continental Ancestry Group" OR "American native continental ancestry group" OR "Asian Continental ancestry group" OR "European Continental Ancestry Group" OR "Oceanic ancestry group" OR "African Americans" OR Arabs
#13 "Asian Americans" OR "Mexican Americans" OR Inuits OR Jews OR "Indians, South American" OR "Indians, North American" OR "South American Indians" OR "North American Indians" OR "Cultural Characteristics"

#14 migrant* OR immigrant* OR asylum OR indig* OR aborig* OR native* OR inuit* OR eskimo* OR kalaallit* OR amerind* OR romany OR romanies OR gypsies OR gipsies OR black OR blacks OR asian OR latin* OR Indian
#15 #11 AND #12 AND #13 AND #14
#16 #5 AND #10 AND #15

WOS:

#1 ALL=("cerebrovascular disorders" OR "basal ganglia cerebrovascular disease" OR "carotid artery thrombosis" OR "carotid artery, internal, dissection" OR "cerebral arterial diseases" OR "cerebral sinus thrombosis" OR "cerebral venous sinus thrombosis" OR "CVST" OR "CVT" OR "anterior cerebral artery infarction" OR "middle cerebral artery infarction" OR "posterior cerebral artery infarction" OR "Lacunar Infarction" OR "intracranial embolism" OR "intracranial thrombosis" OR "brain infarction" OR "vertebral artery dissection" OR "brain ischemia")
#2 TS=((("Ischemi*" OR "Ischaemi*") near/6 ("stroke*" OR "apoplex*" OR "cerebral vasc*" OR "cerebrovasc*" OR cva OR "attack*")))
#3 TS=(((brain OR "cerebr*" OR "cerebell*" OR "vertebrobasil*" OR "hemispher*" OR "intracran*" OR intracerebral OR infratentorial OR supratentorial OR "middle cerebr*" OR "anterior circulation") near/5 ("Ischemi*" OR "Ischaemi*" OR "infarct*" OR "thrombo*" OR "emboli*" OR "occlus*" OR "hypoxi*")))
#4 #1 OR #2 OR #3
#5 ALL=("interventional radiology" OR catheterization OR "balloon catheterization" OR angioplasty OR "laser-assisted balloon angioplasty" OR "laser angioplasty" OR atherectomy OR "catheter ablation" OR embolectomy OR "aspiration embolectomy" OR "balloon embolectomy")
#6 ALL=("penumbra system" OR "solitaire" OR "trevo" OR "neurothrombectom*")
#7 ALL=(thrombectomy OR "blood vessel prosthesis" OR "blood vessel transplantation" OR "cerebral revascularization" OR reperfusion OR dilatation OR angioplast* OR stent* OR atherect* OR thromboaspiration OR endoluminal repair* OR endovascular snare* OR neuronet OR microsnare OR angiojet)
#8 TS=((interventional NEAR/3 ("radiolog*" OR "radiograph*" OR "neuroradiolog*")))
#9 TS=(((mechanical OR "radiolog*" OR pharmacomechanical OR laser OR endovascular OR neurovascular) NEAR/5 ("thrombolys*" OR reperfusion OR fragmentation OR aspiration OR recanalisation OR recanalization OR "clot lysis" OR "clot lyses")))
#10 TS=(((clot OR thrombus OR thrombi OR "embol*") NEAR/5 ("aspirat*" OR "remov*" OR "retriev*" OR fragmentation OR "retract*" OR "extract*" OR "obliterat*" OR "dispers*")))
#11 TS=(((retrieval OR extraction) NEAR/5 "device*"))
#12 TS=((("blood vessel" OR artery) NEAR/5 (prosthesis OR "implantat*")))
#13 TS=(((merci OR concentric) NEAR/5 retriever))
#14 TS=(((Endovascular OR intravascular) NEXT ("Procedure*" OR "Technique*" OR "treatment*")))
#15 #5 OR #6 OR #7 OR #8 OR #9 OR #10 OR #11 OR #12 OR #13 OR #14
#16 ALL=(migrant* OR immigrant* OR refugees OR asylum OR indig* OR aborig* OR native* OR inuit* OR eskimo* OR kalaallit* OR amerind* OR roman* OR gypsies OR gipsies OR black* OR asian OR latin* OR Indian* OR "Mexican Americans" OR Inuits OR Jews OR "Cultural Characteristics" OR "Hispanic Americans" OR "African Continental Ancestry Group" OR "American native continental ancestry group" OR "Asian Continental ancestry group" OR "European Continental Ancestry Group" OR "Oceanic ancestry group" OR "African Americans" OR Arabs OR ethnic* OR race* OR racial OR Nationalit* OR foreign* OR "Minority groups" OR "Ethnic groups" OR Multilingualism OR "Population groups")
#17 #4 AND #15 AND #16

SCOPUS:

(TITLE-ABS-KEY ("cerebrovascular disorders" OR "basal ganglia cerebrovascular disease" OR "brain ischemia" OR "carotid artery diseases" OR "carotid artery thrombosis" OR "carotid artery, internal, dissection" OR "intracranial arterial diseases" OR "cerebral arterial diseases") OR TITLE-ABS-KEY ("anterior cerebral artery infarction" OR "middle cerebral artery infarction" OR "posterior cerebral artery infarction" OR "infarction, anterior cerebral artery" OR "infarction, middle cerebral artery" OR "infarction, posterior cerebral artery") OR TITLE-ABS-KEY ("Lacunar Infarct" OR "Lacunar Infarction" OR "intracranial embolism" OR "intracranial thrombosis" OR "brain infarction" OR "vertebral artery dissection") OR TITLE-ABS-KEY (((brain OR cerebr* OR cerebell* OR vertebrobasil* OR hemispher* OR intracran* OR intracerebral OR infratentorial OR supratentorial OR "middle cerebr*" OR "anterior circulation") W/5 (ischemi* OR ischaemi* OR infarct* OR thrombo* OR emboli* OR occlus* OR hypoxi*))) OR TITLE-ABS-KEY (((ischemi* OR ischaemi*) W/6 (stroke* OR apoplex* OR "cerebral vasc*" OR cerebrovasc* OR cva OR attack*))) OR TITLE-ABS-KEY ("cerebral sinus thrombosis" OR "cerebral venous sinus thrombosis" OR "CVST" OR "CVT"))

AND

(TITLE-ABS-KEY ("interventional radiology" OR catheterization OR "balloon catheterization" OR angioplasty OR "laser-assisted balloon angioplasty" OR "laser angioplasty" OR atherectomy OR "catheter ablation" OR embolectomy OR "aspiration embolectomy" OR "balloon embolectomy") OR TITLE-ABS-KEY (thrombectomy OR "mechanical thrombectomy" OR neurothrombectom* OR "thrombus aspiration" OR "blood vessel prosthesis" OR "blood vessel transplantation" OR "cerebral revascularization" OR reperfusion OR dilatation) OR TITLE-ABS-KEY (angioplast* OR stent* OR atherect* OR thromboaspiration OR endoluminal AND repair* OR endovascular AND snare* OR neuronet OR microsnare OR angiojet OR "penumbra system" OR "solitaire" OR "trevo") OR TITLE-ABS-KEY ((interventional W/3 (radiolog* OR radiograph* OR neuroradiolog*))) OR TITLE-ABS-KEY (((mechanical OR radiolog* OR pharmacomechanical OR laser OR endovascular OR neurovascular) W/5 (thrombolys* OR reperfusion OR fragmentation OR aspiration OR recanalisation OR recanalization OR "clot lysis" OR "clot lyses"))) OR TITLE-ABS-KEY (((clot OR thrombus OR thrombi OR embol*) W/5 (aspirat* OR remov* OR retriev* OR fragmentation OR retract* OR extract* OR obliterat* OR dispers*))) OR TITLE-ABS-KEY (((retrieval OR extraction) W/5 device*)) OR TITLE-ABS-KEY ((("blood vessel" OR artery) W/5 (prosthesis OR implantat*))) OR TITLE-ABS-KEY (((merci OR concentric) near/5 AND retriever)) OR TITLE-ABS-KEY (((endovascular OR intravascular) W/2 (procedure* OR technique* OR treatment*))))

AND

(TITLE-ABS-KEY (ethnic* OR race OR races OR racial OR nationalit* OR foreign* OR "Minority groups" OR "Ethnic groups" OR multilingualism OR refugees OR "Population groups" OR "Continental population groups") OR TITLE-ABS-KEY ("Hispanic Americans" OR "African Continental Ancestry Group" OR "American native continental ancestry group" OR "Asian Continental ancestry group" OR "European Continental Ancestry Group" OR "Oceanic ancestry group" OR "African Americans" OR arabs) OR TITLE-ABS-KEY ("Asian Americans" OR "Mexican Americans" OR inuits OR jews OR "Indians, South American" OR "Indians, North American" OR "South American Indians" OR "North American Indians" OR "Cultural Characteristics") OR TITLE-ABS-KEY (migrant* OR immigrant* OR asylum OR indig* OR aborig* OR native* OR inuit* OR eskimo* OR kalaallit* OR amerind* OR romany OR romanies OR gypsies OR gipsies OR black OR blacks OR asian OR latin* OR indian))
